# *Nigella sativa*: A Potential Antiosteoporotic Agent

**DOI:** 10.1155/2012/696230

**Published:** 2012-09-03

**Authors:** Ahmad Nazrun Shuid, Norazlina Mohamed, Isa Naina Mohamed, Faizah Othman, Farihah Suhaimi, Elvy Suhana Mohd Ramli, Norliza Muhammad, Ima Nirwana Soelaiman

**Affiliations:** Department of Pharmacology, Faculty of Medicine, National University of Malaysia (UKM), Kuala Lumpur Campus, Raja Muda Abdul Aziz Road, 50300 Kuala Lumpur, Malaysia

## Abstract

*Nigella sativa* seeds (NS) has been used traditionally for various illnesses. The most abundant and active component of NS is thymoquinone (TQ). Animal studies have shown that NS and TQ may be used for the treatment of diabetes-induced osteoporosis and for the promotion of fracture healing. The mechanism involved is unclear, but it was postulated that the antioxidative, and anti-inflammatory activities may play some roles in the treatment of osteoporosis as this bone disease has been linked to oxidative stress and inflammation. This paper highlights studies on the antiosteoporotic effects of NS and TQ, the mechanisms behind these effects and their safety profiles. NS and TQ were shown to inhibit inflammatory cytokines such as interleukin-1 and 6 and the transcription factor, nuclear factor **κ**B. NS and TQ were found to be safe at the current dosage for supplementation in human with precautions in children and pregnant women. Both NS and TQ have shown potential as antiosteoporotic agent but more animal and clinical studies are required to further assess their antiosteoporotic efficacies.

## 1. *Nigella sativa*

*Nigella sativa* is a herbal plant which belongs to *Ranunculaceae* family. It is also known as black cumin or *habatus sauda*, and has a rich historical and religious background. It is found in the southern region of Asia. It can grow up to 30 cm and produces pale blue flowers. The fruit is composed of follicles which contain the seeds, the most valuable part of the plant. The seeds of *Nigella sativa* (NS), which have a pungent bitter taste, are used in confectionery and liquors. The seed is the source of the active ingredients of this plant and has been used in Islamic medicine for its healing powers [1]. Studies have revealed various therapeutic values of NS such as anticancer, antioxidant, antibacterial, antifungal, antiparasitic and antiasthmatic [2–7]. NS may also inhibit renal calculi and improve poultry quality [8, 9]. NS contains 36–38% fixed oils, proteins, alkaloids, saponin, and 0.4–2.5% essential oil [10]. High-performance liquid chromatography (HPLC) analysis of NS essential oil revealed that the main active ingredients were thymoquinone, dithymoquinone, thymohydroquinone, and thymol [11]. Among the compounds identified, thymoquinone (TQ) is the most abundant, which makes up 30–48% of the total compounds. This quinine constituent is the most potent and pharmacologically active compound in NS. There were several studies showing that NS and TQ have beneficial effects on bone and joint diseases.

## 2. Osteoporosis

The major bone disease is osteoporosis, a systemic skeletal disease characterized by low bone mass and microarchitectural deterioration of bone tissue, with a consequent increase in bone fragility and susceptibility to fracture. According to World Health Organization (WHO), osteoporosis is defined as a bone mineral density that lays 2.5 standard deviations or more below the average value for young healthy women. In osteoporosis, bone loss occurs especially at the trabecular area when the balance of bone remodeling is tipped towards bone resorption. The bone loss is associated with bone biochemical marker changes such as reduction in osteocalcin level, the marker for bone formation and elevation in cross-link C-telopeptide, the marker for bone resorption. The diagnosis of osteoporosis is made using dual emission X-ray absorptiometry (DEXA) machine but more sophisticated three-dimensional micro-computed tomography (micro-CT) is making way for a better diagnosis.

This paper attempts to discuss the beneficial effects of NS and TQ, its active compound, on osteoporosis and fracture healing.

## 3. Postmenopausal Osteoporosis

The main cause of osteoporosis is menopause or estrogen-deficiency. Several medicinal plants have been studied using postmenopausal osteoporosis animal model such as soy, blueberry, *achyranthes bidentata,* and *labisia pumila* [12–15]. To date, there is no study of NS or TQ on postmenopausal osteoporosis animal model. There was a human study on the effects of NS supplements on the bone markers of postmenopausal women [16]. It was found that NS supplementation for 3 months to these postmenopausal women failed to cause any significant changes in the bone markers levels. The authors concluded that NS was not recommended for the treatment of postmenopausal osteoporosis. However, there were several weaknesses in the study which may account for the nonsignificant results. The sample size of only 15 postmenopausal women was too small. The duration of study should be longer to obtain bone markers readings at several time points and to register any changes in the bone mineral density. Noncompliant in taking NS oil was also a problem due to the nonfavorable greasy taste. In the future, a long term study with larger sample size should be planned. NS in the form of capsules should be given to the study participants to improve compliance. Before that, the effects of NS should be tested in animal osteoporosis models. *In vitro* studies are also required to determine the effects of NS on osteoblasts and osteoclasts.

## 4. Diabetes-Induced Osteoporosis 

Currently, NS has only been tested in animal osteoporosis model of diabetes-induced osteoporosis. No study has been conducted on other osteoporotic animal models. Osteoporosis is now accepted as a major complication of patients with diabetes mellitus. Diabetes could affect bone through multiple mechanisms such as insulin deficiency, insulin resistance, hyperglycemia, or atherosclerosis. However, the exact mechanism responsible for osteopenia in diabetes is still unknown [17]. Insulin and insulin-like growth factors (IGF-1) may have some roles to play in the pathogenesis of diabetic-induced bone loss due to their anabolic effects [18]. 

A study found that combined treatment of NS and parathyroid hormone was more effective in reversing the osteoporotic changes and improving the bone strength of steptozocin-induced diabetic rats than either treatment alone [19]. In another study using the same model, histological assessments found that NS was able to ameliorate diabetic changes of the bone [20]. These findings suggested that NS has potential to be used for the treatment of diabetic osteoporosis.

The mechanisms behind the bone protective effects of NS against diabetes induced-osteoporosis are still unclear. NS may have improved the bone metabolism by improving the blood sugar levels. In certain parts of the world, NS is frequently used as the traditional treatment of diabetes [21]. Studies on streptozotocin-induced diabetic rats have shown that NS may reduce hyperglycaemia, increase serum insulin concentrations, and promote partial regeneration or proliferation of pancreatic beta cells, causing an increase in insulin secretion [22–25]. In other study, NS treatments of diabetic rats have been shown to increase the area of insulin immunoreactive beta-cells. These results have shown that NS may be used as an effective antidiabetic therapy [19]. There is a possibility that NS may have exerted its antiosteoporotic effects in diabetes by improving the blood sugar profile, but further studies are required to confirm this.

Besides that, previous literatures on NS and TQ have highlighted two properties that may be responsible for their antiosteoporotic effects, that is, antoxidative and antiinflammatory properties.

## 5. The Antioxidant Role of NS and TQ against Osteoporosis

Osteoporotic patients were found to be under oxidative stress as their lipid peroxidation levels were elevated and antioxidant enzymes reduced [26, 27]. Most risk factors for osteoporosis were associated with oxidative stress such as hypertension [28], diabetes mellitus [29], and smoking [30]. Exposure to oxidative stress would result in reduction of bone-mineral density [31]. In bone studies, ferric nitril-o-triacetate (FeNTA) was used to induce osteoporosis via oxidative stress [32]. The ferric ions (Fe3+) in FeNTA generate reactive oxygen species through Fenton reaction [33], which may damage bone cells by lipid peroxidation [34, 35]. They could also stimulate osteoclast formation and activity [32, 34, 36], impair osteoblastic function [34, 36], and decrease osteoblast recruitment and collagen synthesis [35]. Free radicals have also been shown to activate nuclear factor-kappa B (NF*κ*B) and raised the levels of bone-resorbing cytokines, interleukin-1(IL-1), and interleukin-6 (IL-6) [32, 37].

Since it is apparent that oxidative stress may lead to osteoporosis, antioxidants may play a role in protecting bone against the damaging effects of free-radicals. Studies have shown that potent anti-oxidants such as tocotrienol and tocopherol, were able to protect bone against FeNTA (oxidative-stress-) induced osteoporosis [32]. 

It is interesting to find that the most significant property of TQ, the active compound of NS, is its antioxidative activities. It has been reported that the freeradical scavenging capability of TQ is as effective as superoxide dismutase [38]. It is most effective in scavenging superoxides, the reactive oxygen species which plays an important role in the activation of osteoclasts [31]. Since TQ is a potent antioxidant, it is expected that it may be able to protect bone against osteoporosis due to oxidative stress. In a cancer study, TQ has been proven to suppress the FeNTA-induced oxidative stress, hyperproliferative response and renal carcinogenesis in rats [39]. In studies using rheumatoid arthritis model, TQ was reported to reduce the serum levels of IL-1 and Tumour Necrosis Factor-*α* [40, 41]. The bone turnover markers, alkaline phosphatase and tartrate-resistant acid phosphatase were also reduced, indicating stable bone formation and resorption activities. The NF*κ*B activation was also blocked by TQ in time-dependent manner [41].

We suspect that the potent antioxidative properties of NS or TQ may account for the antiosteoporotic effects. Furthermore, the antiosteoporotic effects of tocotrienol, another potent antioxidant, have been established in several studies [32, 42]. 

## 6. Anti-Inflammatory Role of NS and TQ in Protecting against Osteoporosis

Inflammation is mediated by two enzymes, cyclooxygenase and lipoxygenase, which generates prostaglandins and leukotrienes from arachidonic acid, respectively [43]. Therefore, both prostaglandins and leukotrienes are the main mediators of inflammation [44]. TQ was believed to exert anti-inflammatory effects by inhibiting the synthesis of prostaglandins and leukotrienes [45, 46]. It was found to inhibit in a dose-dependent manner the cyclooxygenase and lipoxygenase pathways of rat peritoneal leukocytes that were stimulated with calcium ionophore A23187 [47]. 

The antiinflammatory activities of NS were investigated *in vitro*, using the cyclooxygenase (COX) assay. TQ was one of the compounds in NS that was able to inhibit the COX activity at concentrations comparable to indomethacin, a nonsteroidal antiinflammatory drug. Therefore, TQ contributed significantly to the anti-inflammatory activities of NS and has potential to be used as an alternative to nonsteroidal antiinflammatory drugs [48]. Another possible antiinflammatory mechanism of TQ might be suppression of nitric oxide production by macrophages [49]. 

The anti-inflammatory activity of NS was also demonstrated in studies using adjuvant-induced arthritis rat model. Intraperitoneal injections of NS-inhibited carrageenan-induced paw edema in a dose-dependent manner [50]. Similar reduction of formalin-induced paw edema was also observed with oral treatment of NS [51]. 

Osteoporosis is known to be caused by various endocrine, metabolic, and mechanical factors. Recently, plenty of evidences had surfaced, linking inflammation to osteoporosis. This has led to the opinions that inflammation may contribute to osteoporosis [52, 53]. Inflammatory conditions such as ankylosing spondylitis, rheumatoid arthritis and systemic lupus erythematosus were associated with higher incidence of osteoporosis [54–57]. The level of C-reactive protein, a marker of systemic inflammation was also found to be negatively associated with bone mineral density [58]. The extend of osteoporosis is directly related to the degree of inflammation, whereby systemic inflammation resulted in general bone loss, while for local inflammation, bone loss is restricted to the site of inflammation [52]. Elevations of proinflammatory cytokines with aging, gouty arthritis, rheumatoid arthritis and psoriatic arthritis may also contribute to osteoporosis [59–62]. 

**Figure 1:**
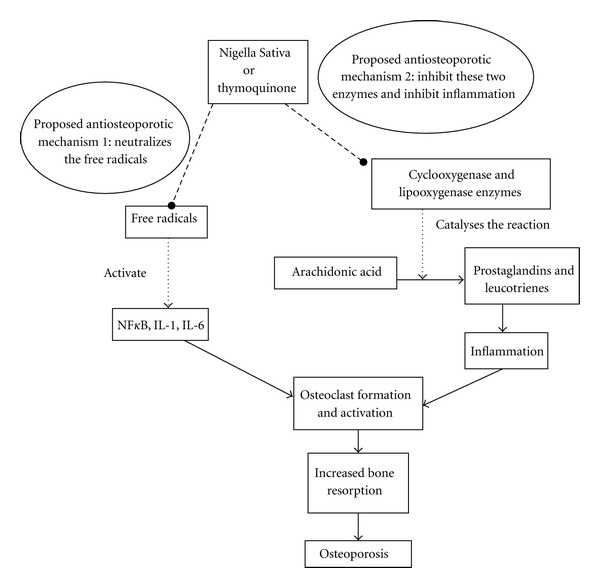
The two pathways which may lead to osteoporosis are shown, that is, activation of osteclastic bone resorption activity by free radicals and by inflammation. The inhibition of these two pathways by *Nigella sativa* or Thymoquinone, its active component, may account for the mechanisms involved in prevention of osteoporosis.

Periodontitis is defined as the inflammation and infection of the ligaments and bones that support the teeth. A study has shown that the alveolar bone loss due to periodontitis was reduced by gastric feeding of TQ to rats. This was accompanied by reduction in osteoclast number and raised osteoblastic activity in TQ-treated rats [63]. The protective effects of TQ on the alveolar bone were thought to be contributed by it antioxidative and anti-inflammatory effects (Figure 1).

## 7. Effects of NS or TQ on Bone Fracture Healing

To date, there is no study on the effects of NS or TQ on the complication of osteoporosis, namely osteoporotic fracture. There is however, an animal study on fracture healing of nonosteoporotic bone, which resembled traumatic fracture healing [64]. In this study, anatomical observations indicated better healing pattern in rats with sustained delivery of TQ. It was concluded that the sustained levels of TQ may enhance bone fracture healing. More studies on fracture healing are required before the effectiveness of NS or TQ in promoting fracture healing can be concluded. In the most recent study, TQ was found to accelerate bone formation and shorten the retention period in rapid maxillary expansion procedure [65].

## 8. Toxicity of NS

As in other herbal preparations, safety is one of the important criteria before NS can be considered for medicinal use. In an acute toxicity study of NS in mice, toxicity signs were first noticed after 4 to 6 hours of NS extract administration. The median lethal dose (LD_50_) was about 470 mg/kg body weight. The toxic signs observed were decreased locomotor activity, decreased sensitivity to touch, and jerking. After 10 hours of administration, the mice exhibited tachypnoea, prostration, and reduced food intake [51]. In other acute and chronic toxicity studies in mice and rats, NS was found to have a wide margin of safety at therapeutic doses. However, attention should be paid to the rise in hematocrit and hemoglobin levels and decrease in white blood cells and platelet counts [66]. 

Sustained delivery of TQ for 30 days using Tri-Calcium Phosphate Lysine (TCPL) capsule loaded with 0.02 grams of TQ to adult male rats have shown little or no side effects on the major vital and reproductive organs [64]. In an *in vitro* study, TQ at the concentration of 0 to 10 *μ*M was not cytotoxic to isolated fibroblast-like synoviocytes [41]. 

The suitable dose for NS extract in human can be extrapolated from animal studies based on the human equivalent dose of no observed adverse effect level (NOAEL) [67]. The human dose is approximately 0.6 mg/kg/day per oral of TQ [41] or 0.05 mL/kg/day per oral of NS extract, which was well received by postmenopausal women [16]. NS extract should be used with precaution in pregnant ladies and children due to its well-known hypoglycemic properties. Diabetic patients should consult with their physicians before taking NS [19, 68]. In children, it was recommended that NS should be administered in weight-adapted doses and given after meals [69]. NS at doses below 80 mg/kg was considered safe in children as there were no adverse effects being reported [70]. 

## 9. Conclusion

NS or TQ has shown potential as a safe and effective antiosteoporotic agent. However, more studies on the effects of NS or TQ using various animal osteoporotic models are required. Once the antiosteoporotic effectiveness of NS or TQ has been established, human studies can be carried out.
